# Benchmarking glycoform-resolved affinity separation – mass spectrometry assays for studying FcγRIIIa binding

**DOI:** 10.3389/fimmu.2024.1347871

**Published:** 2024-02-26

**Authors:** Christoph Gstöttner, Steffen Lippold, Michaela Hook, Feng Yang, Markus Haberger, Manfred Wuhrer, David Falck, Tilman Schlothauer, Elena Domínguez-Vega

**Affiliations:** ^1^ Center for Proteomics and Metabolomics, Leiden University Medical Center, Leiden, Netherlands; ^2^ Protein Analytical Chemistry, Genentech, A Member of the Roche Group, South San Francisco, CA, United States; ^3^ Pharma Technical Development Penzberg, Roche Diagnostics GmbH, Penzberg, Germany; ^4^ Pharma Research and Early Development, Roche Innovation Center, Munich, Germany

**Keywords:** affinity capillary electrophoresis, affinity chromatography, mass spectrometry, FcγRIIIA receptor, monoclonal antibody, glycosylation, affinity interaction

## Abstract

The antibody- FcγRIIIa interaction triggers key immunological responses such as antibody dependent cellular cytotoxicity (ADCC), making it highly important for therapeutic mAbs. Due to the direct glycan-glycan interaction with FcγRIIIa receptor, differences in antibody glycosylation can drastically influence the binding affinity. Understanding the differential binding of mAb glycoforms is a very important, yet challenging task due to the co-existence of multiple glycoforms in a sample. Affinity liquid chromatography (AC) and affinity capillary electrophoresis (ACE) hyphenated with mass spectrometry (MS) can provide glycoform-resolved affinity profiles of proteins based on their differences in either dissociation (AC) or equilibrium (ACE) constants. To cross-validate the affinity ranking provided by these complementary novel approaches, both techniques were benchmarked using the same FcγRIIIa constructs. Both approaches were able to assess the mAb – FcγRIIIa interaction in a glycoform selective manner and showed a clear increase in binding for fully versus hemi-fucosylated mAbs. Also, other features, such as increasing affinity with elevated galactosylation or the binding affinity for high mannose glycoforms were consistent. We further applied these approaches to assess the binding towards the F158 allotype of FcγRIIIa, which was not reported before. The FcγRIIIa F158 allotype showed a very similar profile compared to the V158 receptor with the strongest increase in binding due to afucosylation and only a slight increase in binding with additional galactosylation. Both techniques showed a decrease of the binding affinity for high mannose glycoforms for FcγRIIIa F158 compared to the V158 variant. Overall, both approaches provided very comparable results in line with orthogonal methods proving the capabilities of separation-based affinity approaches to study FcγR binding of antibody glycoforms.

## 1 Introduction

Fcγ receptor IIIa (FcγRIIIa) is a key player in the immune regulation and important mediator of effector functions of therapeutic antibodies, where antibody-dependent cell-mediated cytotoxicity (ADCC) is part of the mechanism of action (1). Two allotypes of FcγRIIIa are present in humans differing by a single amino acid at position 158 (valine or phenylalanine) resulting in the V158 and the F158 variant (2–4). The F158 variant has decreased affinity to IgG1 (5). The resulting FcγRIIIa polymorphism has been correlated with disease susceptibility and efficacy of therapeutic monoclonal antibodies (mAbs) in cancer treatment (6). FcγRIIIa and antibodies are both glycosylated proteins showing a unique glycan-glycan interaction contributing to the binding. The effect of IgG1 Fc glycosylation on FcγRIIIa binding has been studied comprehensively in recent years using a variety of approaches (7–10). Conventional *in-vitro* binding assays for probing the FcγRIIIa-IgG1 interaction are surface plasmon resonance (SPR) or enzyme-linked immunosorbent assays (ELISA) (7). While biophysical *in-vitro* binding assays lack the biological complexity, the binding affinity ranking of monomeric IgG interactions correlates with their biological activity measured by *in-vitro* cellular assays (8, 9). Several reports have consistently demonstrated the effect of overall glycosylation features such as afucosylation, on increased binding to FcγRIIIa (up to 50x increase) (10). However, the influence of other glycan features such as high mannose glycoforms or galactosylation and in particular glycoform pairings, has been difficult to study due to the inability of current binding assays (SPR or ELISA) to distinguish between glycoforms in mixtures. Understanding mAb-receptor binding at the glycoform level is particularly relevant for biopharmaceutical mAbs development, to assist cell line switches and process optimization, batch to batch comparison, or to develop mAbs with enhanced pharmacodynamic behavior. The common strategy to address the binding behavior of mAb glycosylation variants is antibody glycoengineering which often provides an enrichment of glycoforms allowing to establish some trends, but not to assign a binding affinity to each specific mAb glycoform (9, 11, 12).

**Scheme 1:**
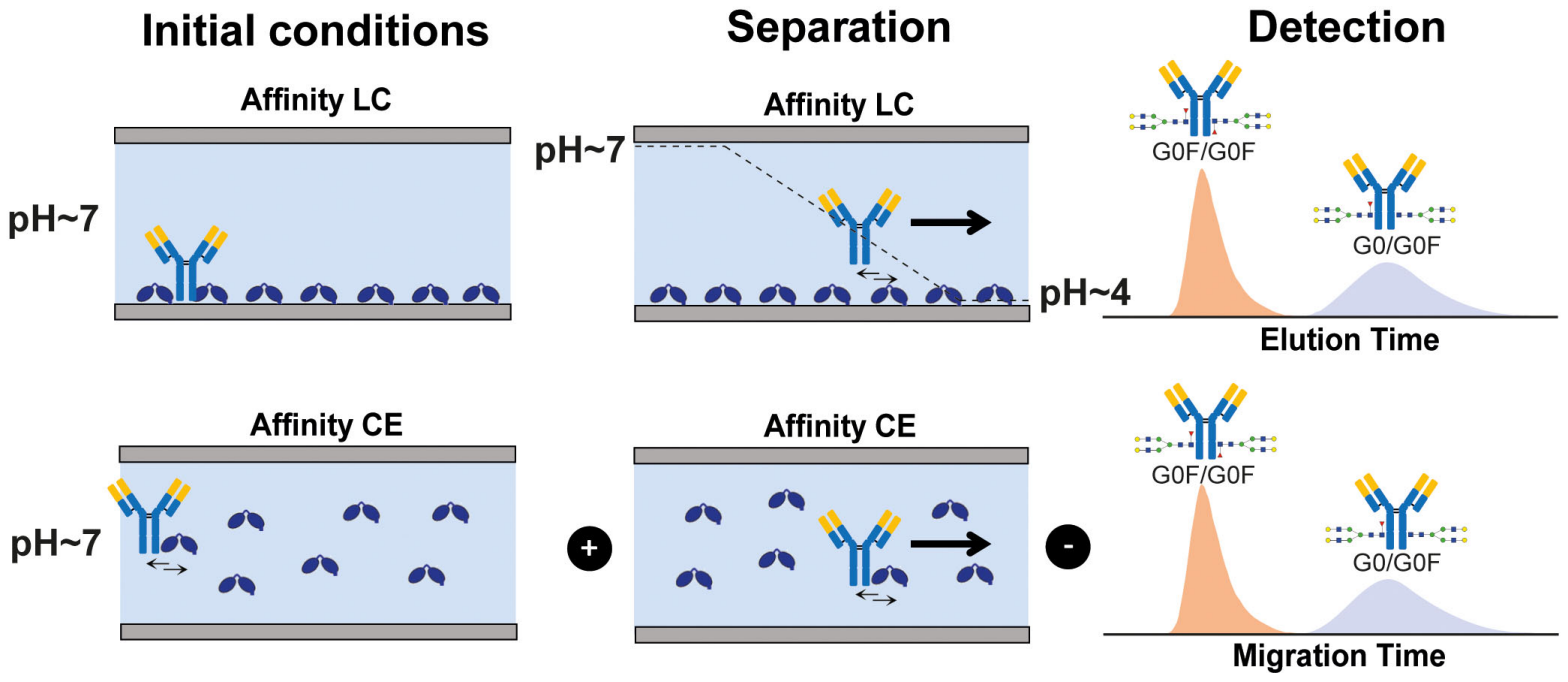
Schematic representation of affinity-based separation techniques AC (upper panel) and ACE (lower panel).

Affinity separation techniques such as affinity capillary electrophoresis (ACE-UV) and affinity chromatography (AC-UV) can determine interactions in protein mixtures for species with large differences in binding and overcome some of these challenges. When hyphenated with mass spectrometry (MS) AC and ACE drastically increase the resolution in protein interaction studies proving particularly useful in revealing small differences in binding affinity between proteoforms (13–16). Both approaches provide detailed information on the affinity ranking of proteoforms in complex mixtures employing different separation mechanisms (**Scheme 1**). Affinity differences are resolved in-solution via an interaction-specific binding equilibrium reflected in the migration time (ACE-MS) or by immobilized ligands and gradual elution (mostly pH changes in AC-MS) reflected in the retention time (AC-MS). The shift in migration time (ACE-MS) or retention time (AC-MS) has been demonstrated to positively correlate with the binding affinity and functional activity.

ACE-MS and AC-MS are powerful platforms for glycoform-resolved interaction studies between IgGs and different FcγRs. We reported the capabilities of both techniques to monitor binding to FcγRIIa by different IgG glycoforms, revealing similar glycoform affinity trends. However, the methods were applied to different mAbs and no direct comparison of both approaches was performed (8, 14). For FcγRIIIa binding, AC-MS has demonstrated to be an excellent tool for dissecting glycoform binding differences (13) but so far no application using ACE-MS has been reported. In this work, we have established a novel FcγRIIIa ACE-MS assay and benchmarked against FcγRIIIa AC-MS using the same IgG1 mAb molecule. The results were compared to conventional orthogonal methods (i.e. SPR, AC-UV and ADCC response) reported in literature using glycoengineered samples. Furthermore, both approaches were applied to the two existing FcγRIIIa allotypes providing the first glycoform-resolved antibody binding comparison between the F158 and the V158 variants.

## 2 Materials and methods

### 2.1 Samples and chemicals

mAb-A as well as the FcγRIIIa purified proteins were kindly provided by Roche Diagnostics (Penzberg, Germany). Both FcγRIIIa purified proteins consisted of the extracellular domain, linked to an AviTag and a LALA-PG mutated IgG1 Fc domain (17). 7.5 M ammonium acetate (AmAc) solution, lysozyme from chicken egg, glacial acetic acid and hydrogen chloride were purchased from Sigma-Aldrich (Steinheim, Germany). Deionized water was obtained using a Milli-Q purification system from EMD Millipore (Burlington, MA). 10 and 30 kDa Vivaspin MWCO filters were purchased from Satorius (Göttingen, Germany).

### 2.2 Affinity capillary electrophoresis – mass spectrometry

For ACE all mAb samples were buffer exchanged to 50 mM AmAc pH 6.8 with a final concentration of 1 μg/μL. FcγRIIIa V158 was buffer exchanged to 50 mM AmAc pH 6.8 and to a final concentration of 0.25 μM. Lysozyme was dissolved in 50 mM AmAc pH 6.8 to a final concentration of 2 μg/μL. For the ACE measurements a Sciex CESI 8000 instrument (Framingham, MA) with a neutrally coated OptiMS capillary (Sciex), equipped with a porous tip, was used. The total/effective length of the capillary was 91cm with an internal diameter of 30 μm and an outer diameter of 150 μm. Before the first use the capillary was flushed for 5 min with 0.1 M HCl (100 psi, forward pressure), followed by 10 min 50 mM AmAc pH 3.0 (100 psi, forward pressure) and 30 min milliQ (100 psi, forward pressure). To ensure proper rehydration of the coating, the capillary was flushed with milliQ for 16h (10 psi, forward pressure). For equilibration before each analysis the capillary was flushed for 2 min with 0.1 M HCl (100 psi, forward pressure), followed by 2 min with milliQ (100 psi, forward pressure) and with 50 mM AmAc pH 6.8 for 2 min forward as well as 2 min reverse pressure of 100 psi. Following the equilibration, the capillary was filled for 2 min with a background electrolyte (BGE) consisting of 50 mM AmAc pH 6.8 containing the FcγRIIIa V158 or F158 at a concentration of 0.25 μM. First a plug of lysozyme was injected for 15 s at a pressure of 1.5 psi followed by the mAb sample which was injected for 15 s at a pressure of 2.5 psi. Finally, a plug of BGE containing the FcγRIIIa V158 was injected at a concentration as mentioned beforehand. The analysis was carried out at 25°C with a constant voltage of 20 kV (normal polarity) with a forward pressure of 2 psi to ensure proper electrospray ionization. At the end of the analysis the voltage was ramped down to 1 kV in 5 min.

For MS detection the CE system was hyphenated using a nano-ESI source to a Exactive Plus EMR from Thermo Fisher Scientific (Waltham, MA). The mass range was set to a range from 1,000 to 15,000 *m/z* with a resolution of 17,500 in positive ionization mode. The In-source CID was set to 200 eV and the collision energy to 75 eV. For each datapoint 10 microscans were acquired with a AGC target of 3e6 and a maximum injection time of 200 ms. The spray voltage was set to 1.7 kV and the capillary temperature to 320°C.

### 2.3 Affinity chromatography – mass spectrometry

The affinity columns were prepared as reported previously (9). In brief, 3 μg/μL of biotinylated FcɣRIIIa were immobilized on streptavidin Sepharose beads (Cytiva) and packed into a Tricorn column housing (Cytiva). All samples were buffer-exchanged (10 kDa MWCO, Merck) to mobile phase A prior to analysis. FcɣRIIIa V158 AC-MS was performed as described elsewhere (13). In short, 50/10ug of mAb were injected to a 1 mL FcγRIIIa 158V/0.5 mL FcγRIIIa 158F column using 50 mM ammonium acetate (pH 5.0 FcγRIIIa V158/pH 6.8 FcγRIIIa F158, mobile phase A) and 50 mM ammonium acetate (pH 3.0 FcγRIIIa V158/pH 4.0 FcγRIIIa F158, mobile phase B). The analysis was performed using a flow-rate of 500 μL/min (FcγRIIIa 158V) or 250 μL/min (FcγRIIIa 158F) and a column temperature of 25°C. Upon binding, isocratic conditions (100% A) were held for 5/2.5 column volumes and then a gradient of 15/7.5 column volumes was applied for elution to 100% B, followed by a 100% B wash for 5/2.5 column volumes and a regeneration step of 15/7.5 column volumes 100% A (V158/F158). UV signals were acquired at 280 nm. Prior to MS, the flow was split to approx. 30 μL/min (FcγRIIIa V158) or 2 μL/min (FcγRIIIa F158) directed to a high-flow ESI Bruker source or Flex ion Thermo source, respectively. FcγRIIIa V158 AC-MS was performed on a 15 T solariX FT-ICR-MS (Bruker Daltonics, Bremen, Germany). The spectra were recorded in a range between 506 to 20,000 *m/z* with 128 k data points and an accumulation time of 1s. For proper declustering of the analyte skimmer 1 was set to 125 V, funnel 1 to 150 V and a radio frequency of 300 Vpp. For ionization a capillary voltage of 4000 V with an endplate offset of −500 V was used. A source temperature of 200°C, nebulizer gas pressure of 0.8 bar and dry gas flow rate of 3 L/min were used. Each spectrum in serial mode analysis resulted from the summation of 20 spectra. This resulted in the acquisition of 2.6 data points per minute in the chromatogram. FcγRIIIa 158F AC-MS was performed on a Q Exactive UHMR Orbitrap (Thermo Scientific). MS data acquisition was performed in positive ion mode (2 kV capillary voltage). The *m/z* range was set from 2,000 Th to 15,000 Th and resolution to 25,000. For improved declustering, desolvation voltage (-175 V) and in-source collision induced dissociation energy (30 V) were applied. For each data point, 10 micro scans were averaged, resulting in a scan rate of 1.6 scans/sec.

### 2.4 Data analysis

Commercial PMI-Byos software (Protein Metrics Inc.; v. 4.5) as well as DataAnalysis (Bruker; v. 5.0) were used for deconvoluting intact mass data. For the PMI intact mass software, a range of charge states from 5+ to 35+ and a mass range from 130 to 160 kDa were applied for deconvolution in relevant retention time windows. In DataAnalysis the deconvolution was performed using the maximum entropy algorithm with a mass range from 130 to 160 kDa, a data point spacing of 1 *m/z* and an instrument resolving power of 5,000. For generation of extracted ion traces (EIC) DataAnalysis was used in the case of measurements performed on the FTICR instrument, whereas Xcalibur or Freestyle (v. 1.8) was used for the generation of EICs for all data measured on Thermo MS instruments. All extracted EICs were smoothed and imported in Adobe illustrator to generate the presented figures.

## 3 Results

### 3.1 ACE- and AC-MS to assess the IgG binding affinity to FcɣRIIIa V158 allotype

**Figure 1:**
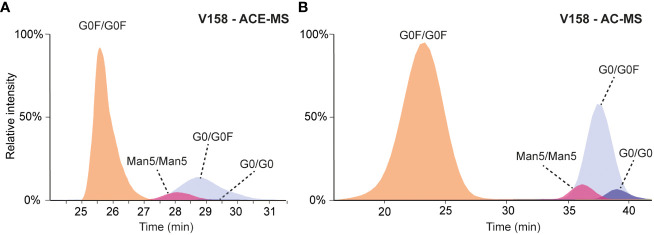
**(A)** Extracted ion chromatograms obtained for the analysis of mAb-A by ACE-MS FcɣRIIIa V158 in the BGE. **(B)** Extracted ion chromatograms obtained for the analysis of mAb-A by AC-MS using a V158 column.

To prove the capabilities and consistency of ACE-MS and AC-MS for glycoform-resolved binding assessment of antibodies, a standard mAb (mAb-A) comprising a mixture of different complex- and high mannose-type glycoforms as well as hemi-glycosylated mAb species (missing one Fc glycosylation) was analyzed using both techniques. ACE and AC can easily be coupled to mass spectrometry by the usage of volatile buffer systems. In both cases, the buffer employed (as BGE or mobile phase for ACE or AC, respectively) consisted of 50 mM ammonium acetate which provides proper ionization while preserving protein conformation (18). For ACE the pH of the BGE was continuously 6.8 during the separation while for AC the binding mobile phase had a pH of 5.0 (or 6.8 for the F158 variant) with an elution buffer towards acidic pH (3.0 for V158 or 4.0 for F158 variant). In both cases, a mass spectrum representative of a native form of the antibody was observed with charge state distributions ranging from 20+ to 27+ charges, when averaging the spectra of the fully fucosylated mAb peak (**Supplementary Figure S1**). Comparing the affinity interaction, both techniques showed a weak binding peak for the fully fucosylated mAb, with an early migration time of 24 to 27 min in case of ACE-MS (**Figure 1A**) or an early elution time of 17 to 23 min in case of AC-MS (**Figure 1B**). The deconvoluted mass spectra of that region revealed a comparable glycoform distribution with observable masses ranging from G0F/G0F to G2F/G2F (**Supplementary Figure S2**). In the deconvoluted spectrum obtained for ACE-MS, additional signals corresponding to the hemi-glycosylated mAbs are visible whereas in the case of AC-MS, hemi-glycosylated mAbs eluted in the isocratic phase (100% Buffer A) indicating very weak affinity under the selected starting conditions. A deconvoluted mass spectra of the peak eluting during the isocratic phase of the AC-MS separation is included in **Supplementary Figure S3** where signals corresponding to the hemi-glycosylated antibody can be observed.

**Figure 2:**
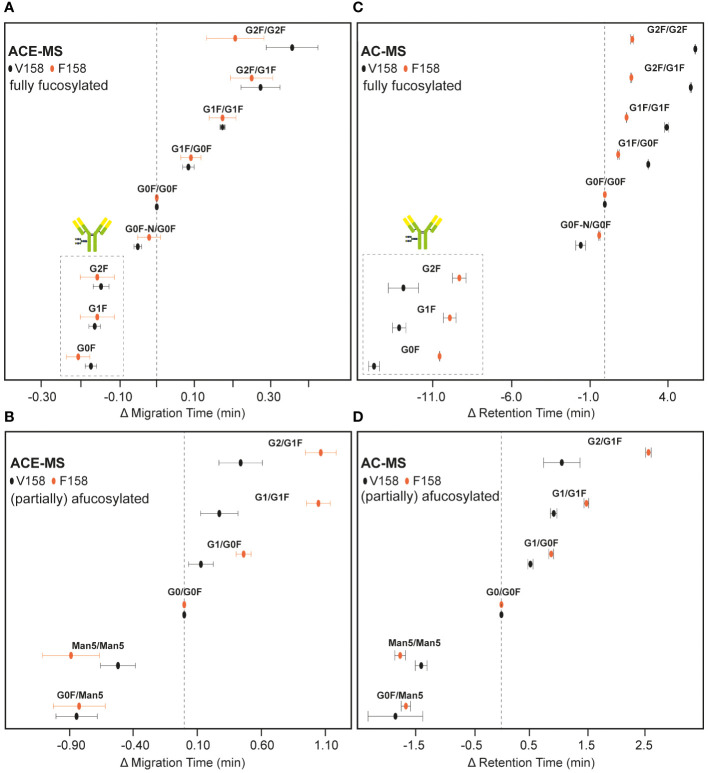
Relative migration and elution time differences of IgG1 mAb glycoforms in ACE-MS and AC-MS. Results obtained by ACE-MS for **(A)** fully fucosylated mAbs or **(B)** hemi-fucosylated and afucosylated mAbs. **(C, D)** represent the corresponding AC-MS measurements. FcɣRIIIa V158 and F158 results are marked by black and orange circles, respectively. The most likely glycoform combinations are given. Insets in **(A, C)** show the hemi-glycosylated mAb species. Differences in retention time of AC-MS for G0F/G0F to G0/G0F are 15.0 min and 8.2 min for V158 and F158, respectively. The difference in migration time of ACE-MS for G0F/G0F to G0/G0F are 2.9 min and 3.0 min for V158 and F158, respectively. Error bars represent the standard error of 3 measurements.

Migrating or eluting later (28-30 min and 35-40 min, respectively), several hemi-fucosylated/afucosylated species were detected indicating higher binding affinity compared to the fully fucosylated mAb variants. For AC-MS the resolution between the fully fucosylated and the (partially) afucosylated species was higher than for ACE-MS. Due to the asymmetric binding of the FcɣRIIIa, the lack of one fucose on one side of the IgG molecule is already enough to increase the affinity (19, 20). The additional loss of the second fucose only resulted in a slight increase of the binding to FcɣRIIIa. This effect was consistently observed for both affinity techniques. Antibodies with a Man5/Man5 glycosylation showed a lower binding affinity compared to the mAb containing afucosylated complex-type glycans, yet stronger than the fully fucosylated mAb. The effect of other antibody glycan features such as galactosylation in binding to FcɣRIIIa are represented in **Figure 2**, where G0F/G0F for fully fucosylated species and G0/G0F for (partially) afucosylated species was used as a reference point. Plotting the relative changes in retention/migration time allows an easy comparison between different techniques and receptors even though the actual retention/migration times show larger differences (see **Supplementary Tables S1, S2**). These plots show that increasing galactosylation levels resulted in an increase of migration/elution time indicating a positive influence on binding (**Figure 2**). This effect was observed for fucosylated (**Figures 2A, C**, **Supplementary Table S1, S2**) and afucosylated variants (**Figures 2B, D**, **Supplementary Table S1, S2**) and was consistent for both techniques. The mAb containing only one Man5 glycosylation paired with G0F showed a lower binding affinity compared to the Man5/Man5 mAb. Additionally, in the case of the fully fucosylated mAb the lack of one terminal *N*-acetylglucosamine resulted in a slight decrease in binding affinity compared to the G0F/G0F mAb. Overall, the elution order observed with both affinity separation approaches correlated well between both techniques (**Supplementary Figure S4**) providing similar information on the relative binding of mAb glycoforms.

### 3.2 ACE- and AC-MS to assess the IgG binding affinity to FcɣRIIIa F158 allotype

**Figure 3:**
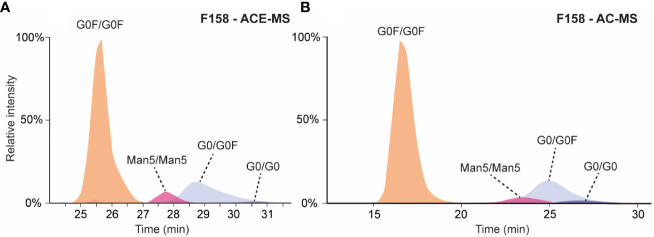
**(A)** Extracted ion chromatograms obtained for the analysis of mAb-A by ACE-MS with FcɣRIIIa F158 in the BGE. **(B)** Extracted ion chromatograms obtained for the analysis of mAb-A by AC-MS using a F158 column.

Both affinity separation techniques were applied to assess the binding affinity of antibody glycoforms to the second FcɣRIIIa allotype containing a phenylalanine in position 158 instead of a valine. Previous studies analyzing antibody mixtures by SPR resulted in K_D_ values for a wildtype IgG1 Fc between 2 and 3 µM for F158 and around 0.4 µM for the FcɣRIIIa V158 (21, 22). Whereas ACE-MS could cope with this difference and the same experimental conditions could be employed for the analysis of mAbs with FcɣRIIIa V158 and F158, AC-MS needed some adaptations in the mobile phase composition and elution gradient. Therefore, the pH of the mobile phase was increased from 5 to 6.8 for the binding mobile phase (A) and from 3.0 to 4.0 for the elution mobile phase (B) to provide similar elution profile than the FcɣRIIIa V158 variant (**Figures 1**, **3**).

In both cases (AC-MS and ACE-MS) the affinity separation order for the fully fucosylated, hemi-fucosylated and afucosylated mAbs was comparable to the V158 variant (**Figure 3**). The relative affinity of different fully fucosylated as well as hemi-glycosylated mAbs in comparison to the mAb containing G0F/G0F was determined and illustrated in **Figure 2**, for AC-MS and ACE-MS and both receptor allotypes. In **Figures 2B, D** we assessed the binding affinity of hemi-fucosylated or afucosylated mAb glyco-variants compared to the G0/G0F mAb. A larger decrease in binding of the Man5/Man5 species relative to the G0/G0F species was observed with both approaches for FcɣRIIIa F158 compared to FcɣRIIIa V158 (**Figure 2**). In contrast, there was no difference observed for G0F/Man5 between both receptor variants. Overall galactosylation showed a positive influence on the binding to the FcɣRIIIa receptor for both variants. However, it seems that for the fully fucosylated mAbs higher galactosylation shows an increased affinity difference to the FcɣRIIIa V158 receptor compared to the F158 allotype, whereas for the hemi-fucosylated species the opposite effect can be observed with a stronger affinity of higher galactosylated species to the F158 receptor variant. This trend is very similar for both affinity methods and most dominant for the G2F/G2F and the G2F/G1F, as well as for the G1/G1F and G2/G1F in case of the hemi-fucosylated mAbs. Furthermore, for the G0F-N/G0F mAb only a slight influence in the affinity to the F158 in comparison to G0F/G0F can be observed, whereas for the V158 allotype shows a larger decrease in affinity.

Additionally, biopharmaceuticals can contain some amounts of hemi-glycosylated antibodies, which were linked to lower ADCC activities. In the case of ACE-MS we are able to assess the binding affinity for these species resulting in a slightly lower affinity compared to G0F/G0F species (**Figures 2A, C**, insets). In the case of AC-MS these species elute before the pH gradient due to their low interaction with the FcɣRIIIa which shows that for AC small adjustments are needed to obtain good coverage of the whole affinity range.

## 4 Discussion

Glycoform-selective binding assessment of antibodies and FcɣRIIIa receptors is an important, yet tedious process due to the lack of selectivity to distinguish between co-existing species of most binding techniques. For instance, in the case of SPR and ELISA the heterogeneity in the binding strength of different mAb glycoforms within the same sample lead to a competitive binding to the Fc receptor resulting in an overrepresentation of afucosylated mAbs. Here, we benchmarked two promising and novel affinity-separation techniques for glycoform-selective binding assessment of mAbs towards the FcɣRIIIa receptor. For both techniques ammonium acetate was used as a buffer allowing hyphenation to mass spectrometry. In the case of ACE, the same experimental conditions could be applied to different FcɣRIIIa variants without the need for adaptation. In contrast, for AC-MS experiments the mobile phases and gradient needed minor adaptation within receptor variants to enable a binding to the receptor. Another important consideration in AC-MS is the potential change in interaction properties between mAb and Fc receptor under low pH values as well as a negative impact on the column stability. Both approaches provide information in relative binding affinity due to differences in the elution/migration pattern. The core difference between these approaches relies on the immobilization of the receptor in the stationary phase for AC-MS and determination of binding affinity by dissociation, while for ACE-MS the receptor constructs are added to the separation buffer and are free in solution during the analysis. The binding affinity in ACE-MS is determined by an equilibrium in binding and dissociation reflected in a temporary complexation of the antibody with the receptor resulting in a shift of the electrophoretic mobility. This introduces complementarity which can be exploited to choose the optimal technique depending on the application. ACE-MS consumes only minor amounts of receptor (µgs) being ideal for testing of receptor variants where limited amounts are available. AC-MS often needs higher amounts of receptor (few mg) and requires immobilization to a stationary phase and labor-intensive column packing, but once ready, the column (and receptor) can be re-used, being more suitable for hundreds of analyses. In ACE-MS, the shift of the electrophoretic mobility in absence and presence of receptor are used for the affinity measurements. Therefore, intrinsic differences in the electrophoretic mobility of different molecules are corrected, enabling the simultaneous application to multiple antibodies. On the contrary, AC-MS relies on the differences in retention and molecules with different physicochemical characteristics can be retained differently (23), thus limiting the application to glycoforms of the same antibody. In addition, due to the absence of stationary phase, potential secondary interactions of the IgG are avoided in ACE-MS compared to AC-MS.

Besides the different experimental and operational set-ups, both techniques provided comparable results in terms of order and qualitative extent of affinity differences. In AC-MS, the separation is dependent on the mobile phase composition and elution gradient, permitting to optimize the conditions to maximize the resolution between species. As in ACE-MS the separation is based on the interaction at equilibrium, there is not elution phase and the BGE options are largely restricted due to the native/volatile requirements. Therefore, AC-MS provided higher resolution between species and therefore, it was more sensitive to minor binding differences (e.g. galactosylation). Furthermore, AC-MS showed overall lower variation in elution time compared to migration time in ACE-MS (**Figure 2**), making the AC-MS platform more suitable for batch-to-batch comparison where the same IgG antibody needs to be analyzed repeatedly. ACE-MS has the benefit that different receptors and antibodies can be examined very quickly by omitting the need to pack receptor columns or adjust gradients for different mAb molecules, which, in combination with the low protein consumption, makes it a perfect tool for the early research stage of mAbs or new mAb formats.

Regarding the affinity differences of the examined antibody, in both cases the main contributor to the affinity was the lack of one or two fucoses in the antibody. These results match very well with results obtained by AC-UV showing strong increase in binding with (partial) afucosylated mAbs as well as an increased ADCC (9, 11, 12, 24, 25). For galactosylation a small, yet visible, increase in affinity was observed with increasing number of galactose molecules, which was also observed previously by SPR as well as NK cell-mediated induction of ADCC, where glycoengineered antibodies with high galactosylation levels showed higher binding affinity than antibodies with low galactosylation levels (9, 11, 12, 25). Recently, it was shown that the Fc can adopt different conformational states (open versus closed) depending on the glycosylation, with afucosylation resulting in an open state (26). Therefore, it is likely that an increase in galactosylation leads to a more open state allowing a better interaction between IgG and FcɣRIIIa, explaining the increased binding affinity. Next to complex type glycans, high mannose species were monitored. Man5/Man5 as well as G0F/Man5 showed an increased affinity compared to fucosylated complex-type glycans, yet lower than the antibody with afucosylated glycans. The increase in binding affinity of high mannose glycoforms to the FcɣRIIIa compared to the fully fucosylated mAb is also supported by previous reports showing an increased ADCC response and binding to the FcɣRIIIa, where glycoengineered antibodies were compared to mAbs with wildtype glycosylation (12, 25, 27).

To obtain some insight in the binding behavior of the FcɣRIIIa F158 allotype in a glycoform specific manner for the first time, we analyzed it in parallel to the V158 variant. Similar to the V158 variant, we observed a strong increase in binding affinity with the lack of one core fucose, which aligns with results in literature analyzing a glycoengineered mAb by SPR (11). Whereas the effect on the affinity of G0F/Man5 was very similar for both receptor variants (V158 and F158), mAbs containing Man5/Man5 showed a lower binding affinity to the FcɣRIIIa F158. Interestingly, the effect of additional galactoses on fully fucosylated mAbs was also slightly different between receptor variants with a lower increase in binding affinity in the case of the FcɣRIIIa F158 variant. However, this effect was reversed for a hemi-fucosylated mAb with a higher increase in binding affinity to the FcɣRIIIa F158 per galactose added compared to the V158 variant. A similar result was reported by Dekkers et al. using glycoengineered samples containing low levels of fucose where an increase of galactosylation showed a stronger influence on the binding to the F158 variant compared to the V158 receptor variant (11). A possible explanation of the differential binding behavior could be the exchange of the small amino acid valine by the larger tryptophan in the IgG binding site. This exchange leads to some small alterations in the FcɣRIIIa structure (28, 29) which might not only cause the overall lower affinity but also the changed response to different IgG glycosylation. Hemi-glycosylated mAbs showed slightly lower binding affinity compared to the G0F/G0F mAb. The relative binding of these species compared to fully glycosylated antibodies could not be assessed by AC-MS due to the elution of these forms during the isocratic binding phase (i.e. elution under different experimental conditions) yet differences in binding within different galactosylation levels of hemi-glycosylated species were observed.

Overall, both approaches demonstrated to be very powerful in the assessment of binding affinities between mAbs and Fc receptors allowing an extended functional characterization. One benefit is the omission of tedious production of highly pure glycoforms by glycoengineering as it was required when using conventional binding techniques or by employing UV detection instead of MS. AC-MS and ACE-MS have not only the potential to study different glycosylation levels, but can in the future also be extended to assess other post translational modifications which impact the binding to Fc receptors. This would allow to establish a structure-function relationship in a proteoform-resolved manner. Overall ACE-MS can be seen as a tool to screen different mAb molecules or receptor variants in a short time frame or if material is scarce. AC-MS, on the other hand, with its robust elution time and user-friendliness is the perfect tool to assess the affinity of different mAbs in a quality control setting where several samples from the same mAb, such as production batches, need to be analyzed. In addition, AC can be used in a small preparative scale to collect samples for orthogonal assessment. Receptor glycosylation can also influence the binding affinity, however, very little is known about the differential binding of FcɣRIIIa glycoforms. The proposed approaches offer great opportunities to study the influence of receptor glycan heterogeneity in binding affinity and will be the matter of future studies in our lab.

## Data availability statement

The original contributions presented in the study are included in the article/**Supplementary Material**. Further inquiries can be directed to the corresponding author.

## Author contributions

CG: Conceptualization, Data curation, Formal analysis, Investigation, Methodology, Writing – original draft, Writing – review & editing. SL: Data curation, Formal analysis, Investigation, Writing – review & editing. MiH: Writing – review & editing. FY: Writing – review & editing. MaH: Resources, Writing – review & editing. MW: Writing – review & editing, Funding acquisition. DF: Writing – review & editing. TS: Writing – review & editing, Conceptualization, Resources. ED-V: Conceptualization, Writing – review & editing, Funding acquisition, Investigation, Methodology, Supervision, Writing – original draft.
