# Supplementary material for: Benchmarking glycoform-resolved affinity separation – mass spectrometry assays for studying FcγRIIIa binding
Source: Front Immunol. 2024 Feb 26;15:1347871. doi: 10.3389/fimmu.2024.1347871 (PMC10925690; doi:10.3389/fimmu.2024.1347871)
Supplement: Supplementary file 1 [file DataSheet_1.docx]

**Supplementary Information**

**Benchmarking glycoform-resolved affinity separation – mass spectrometry assays for studying FcγRIIIa binding**

**Christoph Gstöttner^1^, Steffen Lippold^2^, Michaela Hook^3^, Feng Yang^2^, Markus Haberger^3^, Manfred Wuhrer^1^, David Falck^1^, Tilman Schlothauer^4^, Elena Domínguez-Vega^1^***

^1^Leiden University Medical Center, Center for Proteomics and Metabolomics, Leiden, The Netherlands.

^2^Protein Analytical Chemistry, Genentech, A Member of the Roche Group, South San Francisco, CA, United States.

^3^Pharma Technical Development Penzberg, Roche Diagnostics GmbH, Penzberg, Germany.

^4^Pharma Research and Early Development, Roche Innovation Center Munich, Germany.

*** Correspondence:**Elena Domínguez-Vega
e.dominguez_vega@lumc.nl


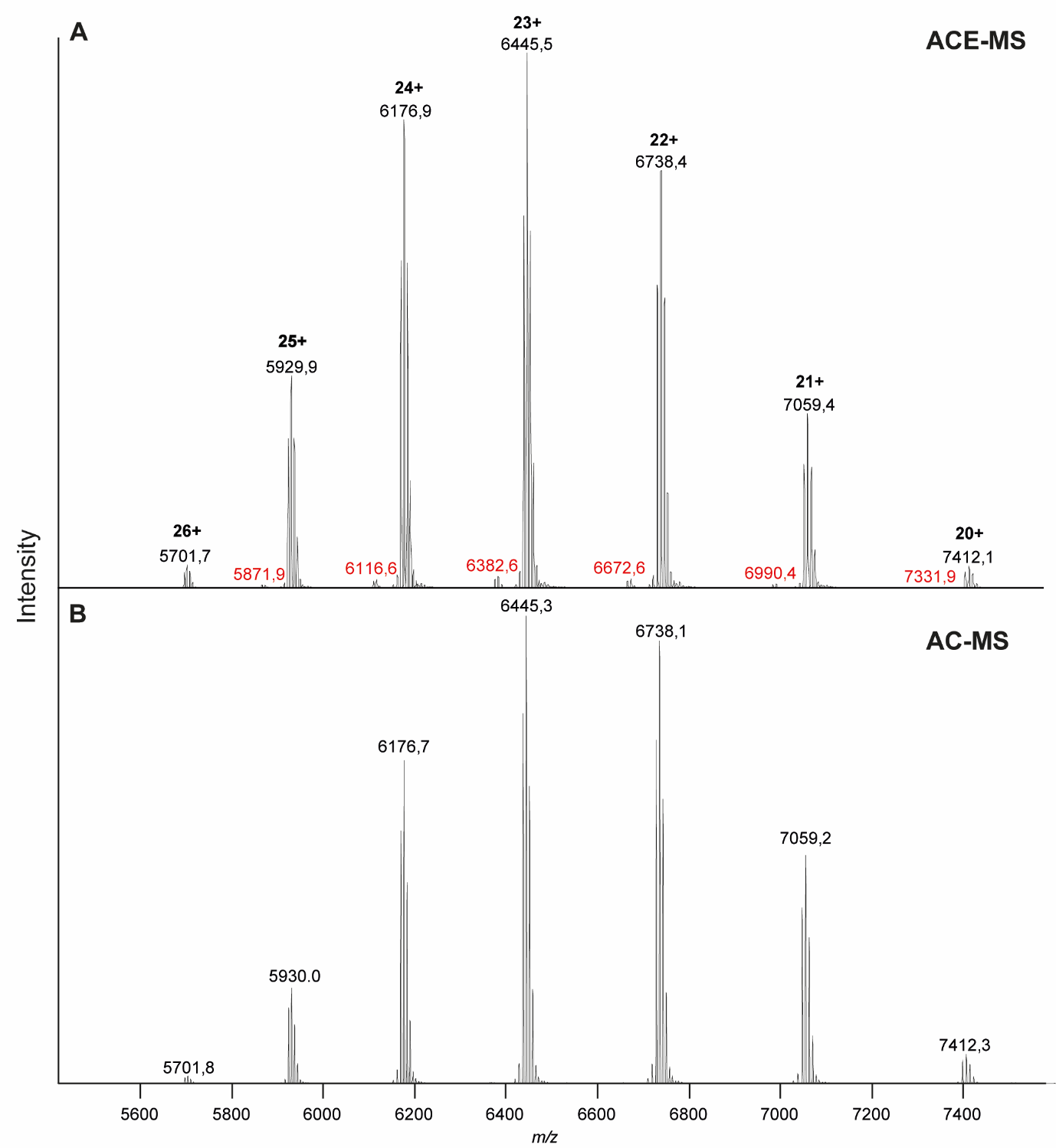


**Figure S1.** Comparison of the mass spectra of the peak containing the fully fucosylated mAb obtained by ACE-MS (A) and by AC-MS (B). The annotation of the signals correspond to the m/z and the corresponding charge state. The red numbers in case of ACE-MS represent the hemi-glycosylated mAb signals.

**
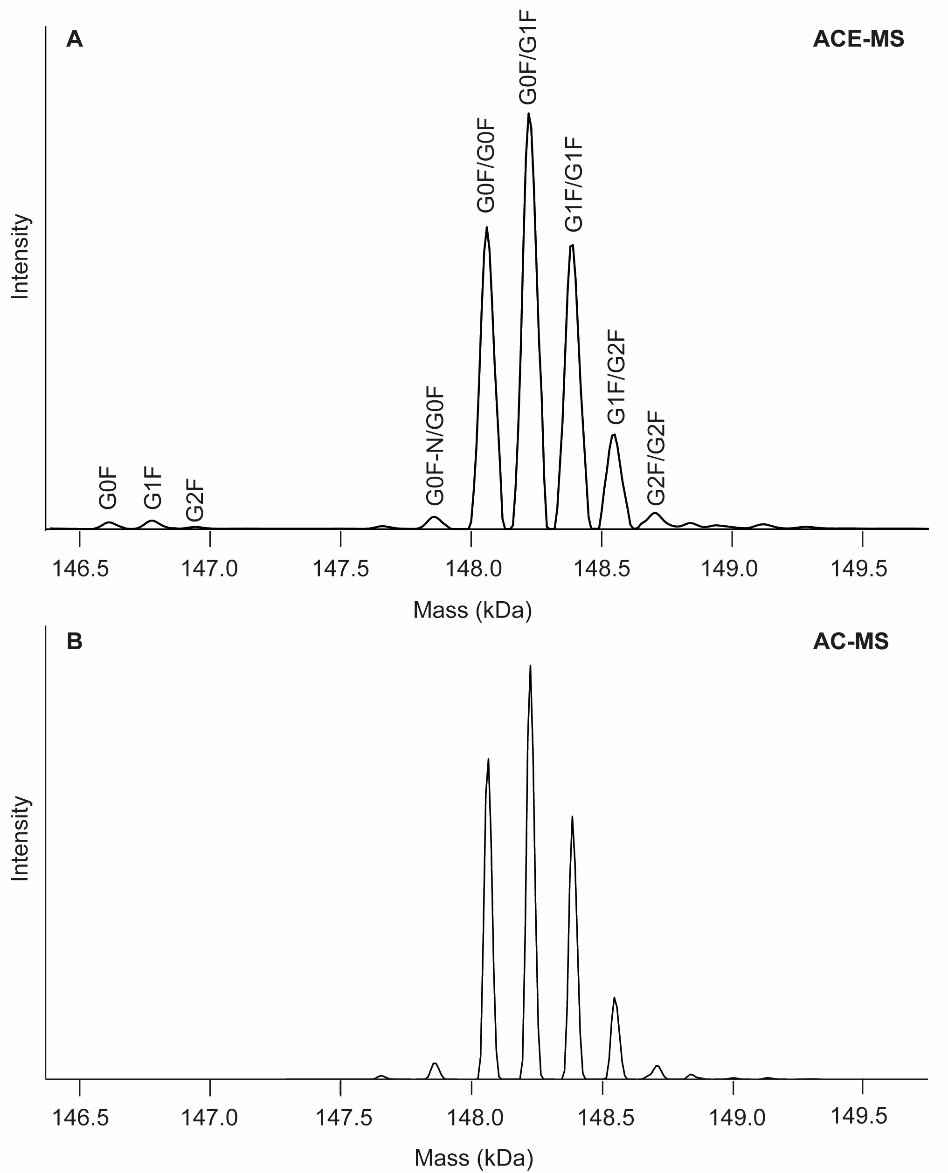
**

**Figure S2.** Comparison of the deconvoluted mass spectra of the peak containing the fully fucosylated mAb obtained by ACE-MS (A) and AC-MS (B). For the antibody carrying the two glycans, the peaks are labeled with the most likely combination of glycoforms.

**
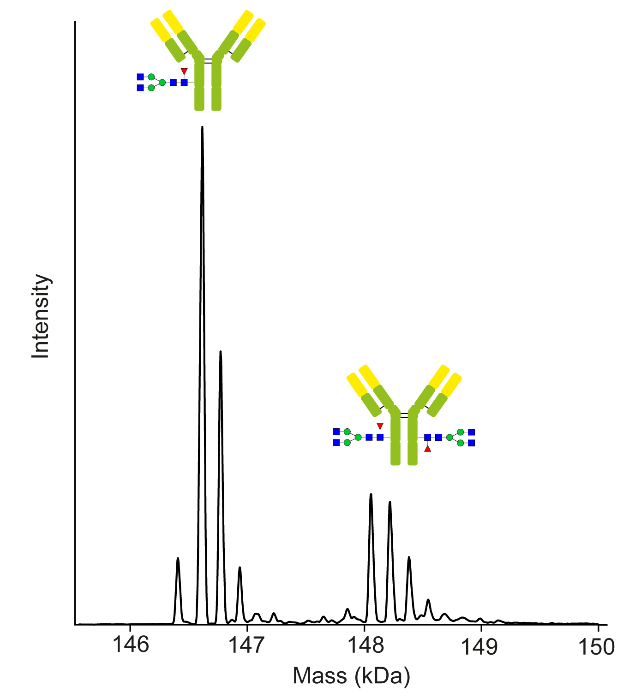
**

**Figure S3.** Deconvoluted mass spectrum of the peak eluted during the isocratic phase (100% Buffer A) of the AC-MS separation.


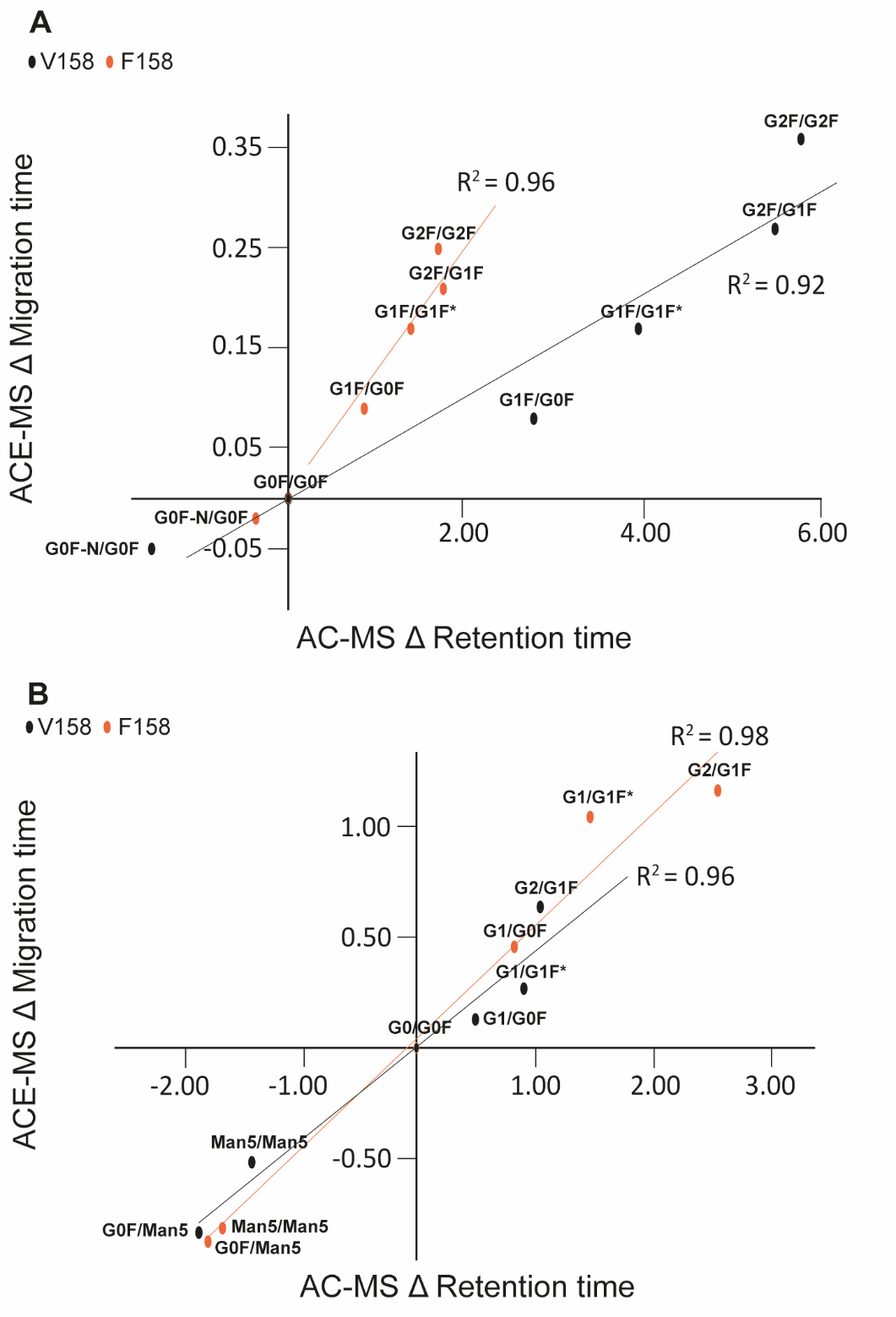


**Figure S4**. Correlation between ACE-MS and AC-MS for the fully fucosylated mAb glyco-variants (A) and the hemi-fucosylated or afucosylated mAb glyco-variants (B).

|  | **V158** | | | | | **F158** | | | | |
| --- | --- | --- | --- | --- | --- | --- | --- | --- | --- | --- |
| **Glycoform** | **Replicate 1** | **Replicate 2** | **Replicate 3** | **Average** | **Deviation** | **Replicate 1** | **Replicate 2** | **Replicate 3** | **Average** | **Deviation** |
| **G0F/G0F** | 0.00 | 0.00 | 0.00 | 0.00 | 0.00 | 0.00 | 0.00 | 0.00 | 0.00 | 0.00 |
| **G0F-N/G0F** | -0.06 | -0.04 | -0.05 | -0.05 | 0.01 | 0.01 | -0.02 | -0.05 | -0.02 | 0.03 |
| **G0F/G1F** | 0.07 | 0.10 | 0.08 | 0.08 | 0.02 | 0.08 | 0.12 | 0.07 | 0.09 | 0.03 |
| **G1F/G1F (G0F/G2F)** | 0.18 | 0.17 | 0.17 | 0.17 | 0.01 | 0.17 | 0.21 | 0.14 | 0.17 | 0.04 |
| **G1F/G2F** | 0.33 | 0.23 | 0.26 | 0.27 | 0.05 | 0.26 | 0.3 | 0.19 | 0.25 | 0.06 |
| **G2F/G2F** | 0.38 | 0.28 | 0.41 | 0.36 | 0.07 | 0.26 | 0.24 | 0.12 | 0.21 | 0.08 |
| **G0F** | -0.19 | -0.17 | -0.16 | -0.17 | 0.02 | -0.24 | -0.2 | -0.18 | -0.21 | 0.03 |
| **G1F** | -0.18 | -0.16 | -0.15 | -0.16 | 0.02 | -0.2 | -0.11 | -0.16 | -0.16 | 0.05 |
| **G2F** | -0.17 | -0.14 | -0.13 | -0.15 | 0.02 | -0.2 | -0.11 | -0.16 | -0.16 | 0.05 |
| **G0/G0F** | 0.00 | 0.00 | 0.00 | 0.00 | 0.00 | 0.00 | 0.00 | 0.00 | 0.00 | 0.00 |
| **G1/G0F (G0/G1F)** | 0.09 | 0.24 | 0.06 | 0.13 | 0.10 | 0.44 | 0.42 | 0.53 | 0.46 | 0.06 |
| **G1/G1F (G0/G2F, G0F/G2)** | 0.44 | 0.21 | 0.17 | 0.27 | 0.15 | 1.1 | 1.1 | 0.94 | 1.05 | 0.09 |
| **G1/G2F (G1F/G2)** | 0.56 | n.d. | 0.32 | 0.44 | 0.17 | 1.15 | n.d. | 0.98 | 1.07 | 0.12 |
| **G0F/Man5** | -0.99 | -0.67 | -0.86 | -0.84 | 0.16 | -1.04 | -0.64 | -0.78 | -0.82 | 0.20 |
| **Man5/Man5** | -0.47 | -0.41 | -0.67 | -0.52 | 0.14 | -1.09 | -0.65 | -0.91 | -0.88 | 0.22 |

**Table S1**: Retention time shifts obtained for three ACE-MS measurements using either the V158 or F158 variant.

|  | *V158* | | | | | | *F158* | | | | | |
| --- | --- | --- | --- | --- | --- | --- | --- | --- | --- | --- | --- | --- |
| *Glycoform* | **Replicate 1** | **Replicate 2** | **Replicate 3** | **Average** | **Deviation** | **Replicate 1** | | **Replicate 2** | **Replicate 3** | **Average** | **Deviation** |  |
| *G0F/G0F* | 0.00 | 0.00 | 0.00 | 0.00 | 0.00 | 0.00 | | 0.00 | 0.00 | 0.00 | 0.00 |  |
| *G0F-N/G0F* | -1.19 | -1.65 | -1.78 | -1.54 | 0.31 | -0.34 | | -0.4 | -0.35 | -0.36 | 0.03 |  |
| *G0F/G1F* | 2.77 | 2.76 | 2.76 | 2.76 | 0.01 | 0.85 | | 0.86 | 0.85 | 0.85 | 0.01 |  |
| *G1F/G1F (G0F/G2F)* | 3.86 | 4.11 | 3.85 | 3.94 | 0.15 | 1.34 | | 1.35 | 1.45 | 1.38 | 0.06 |  |
| *G1F/G2F* | 5.51 | 5.49 | 5.43 | 5.48 | 0.04 | 1.69 | | 1.66 | 1.72 | 1.69 | 0.03 |  |
| *G2F/G2F* | 5.74 | 5.78 | 5.79 | 5.77 | 0.03 | 1.8 | | 1.7 | 1.73 | 1.74 | 0.05 |  |
| *G0F* | -15.07 | -14.38 | -14.71 | -14.72 | 0.35 | -10.57 | | -10.57 | -10.48 | -10.54 | 0.05 |  |
| *G1F* | -12.66 | -13.18 | -13.51 | -13.12 | 0.43 | -10.39 | | -9.66 | -9.62 | -9.89 | 0.43 |  |
| *G2F* | -13.87 | -11.97 | -12.7 | -12.85 | 0.96 | -9.04 | | -9.81 | -9 | -9.28 | 0.46 |  |
| *G0/G0F* | 0.00 | 0.00 | 0.00 | 0.00 | 0.00 | 0.00 | | 0.00 | 0.00 | 0.00 | 0.00 |  |
| *G1/G0F (G0/G1F)* | 0.55 | 0.5 | 0.46 | 0.50 | 0.05 | 0.89 | | 0.83 | 0.83 | 0.83 | 0.00 |  |
| *G1/G1F (G0/G2F, G0F/G2)* | 0.97 | 0.89 | 0.86 | 0.91 | 0.06 | 1.53 | | 1.44 | 1.44 | 1.44 | 0.00 |  |
| *G1/G2F (G1F/G2)* | 1.35 | 1.07 | 0.72 | 1.05 | 0.32 | 2.52 | | 2.6 | 2.6 | 2.60 | 0.00 |  |
| *G0F/Man5* | -1.62 | -1.51 | -2.38 | -1.84 | 0.47 | -1.72 | | -1.64 | -1.64 | -1.64 | 0.00 |  |
| *Man5/Man5* | -1.28 | -1.41 | -1.48 | -1.39 | 0.10 | -1.71 | | -1.85 | -1.85 | -1.85 | 0.00 |  |

**Table S2**: Retention time shifts obtained for three AC-MS measurements using either the V158 or F158 variant.
